# Global REACH: Assessment of Brady-Arrhythmias in Andeans and Lowlanders During Apnea at 4330 m

**DOI:** 10.3389/fphys.2019.01603

**Published:** 2020-01-22

**Authors:** Stephen A. Busch, Sean van Diepen, Andrew R. Steele, Victoria L. Meah, Lydia L. Simpson, Rómulo J. Figueroa-Mujíca, Gustavo Vizcardo-Galindo, Francisco C. Villafuerte, Michael M. Tymko, Philip N. Ainslie, Jonathan P. Moore, Mike Stembridge, Craig D. Steinback

**Affiliations:** ^1^Neurovascular Health Lab, Faculty of Kinesiology, Sport, and Recreation, University of Alberta, Edmonton, AB, Canada; ^2^Department of Critical Care and Division of Cardiology, Department of Medicine, University of Alberta, Edmonton, AB, Canada; ^3^School of Sport, Health and Exercise Sciences, Bangor University, Bangor, United Kingdom; ^4^Laboratorio de Fisiología Comparada, Departamento de Ciencias Biológicas y Fisiológicas, Facultad de Ciencias y Filosofía, Universidad Peruana Cayetano Heredia, Lima, Peru; ^5^Centre for Heart, Lung and Vascular Health, University of British Columbia Okanagan, Kelowna, BC, Canada; ^6^Cardiff Centre for Exercise and Health, Cardiff School of Sport and Health Sciences, Cardiff Metropolitan University, Cardiff, United Kingdom

**Keywords:** hypoxia, Andean, arrhythmia, chronic mountain sickness, electrophysiology, high altitude physiology, cardiac

## Abstract

**Background:** Ascent to altitude increases the prevalence of arrhythmogenesis in low-altitude dwelling populations (Lowlanders). High altitude populations (i.e., Nepalese Sherpa) may have arrhythmias resistant adaptations that prevent arrhythmogenesis at altitude, though this has not been documented in other High altitude groups, including those diagnosed with chronic mountain sickness (CMS). We investigated whether healthy (CMS-) and CMS afflicted (CMS +) Andeans exhibit cardiac arrhythmias under acute apneic stress at altitude.

**Methods and Results:** Electrocardiograms (lead II) were collected in CMS- (*N* = 9), CMS + (*N* = 8), and Lowlanders (*N* = 13) following several days at 4330 m (Cerro de Pasco, Peru). ECG rhythm and HR were assessed at both rest and during maximal volitional apnea. Both CMS- and CMS + had similar basal HR (69 ± 8 beats/min vs. 62 ± 11 beats/min), while basal HR was higher in Lowlanders (77 ± 18 beats/min; *P* < 0.05 versus CMS +). Apnea elicited significant bradycardia (nadir −32 ± 15 beats/min; *P* < 0.01) and the development of arrhythmias in 8/13 Lowlanders (junctional rhythm, 3° atrio-ventricular block, sinus pause). HR was preserved was prior to volitional breakpoint in both CMS- (nadir −6 ± 1 beat/min) and CMS + (1 ± 12 beats/min), with 2/17 Andeans developing arrhythmias (1 CMS+ and 1 CMS-; both Premature atrial contraction) prior to breakpoint.

**Conclusion:** Andeans showed an absence of arrhythmias and preserved HR response to volitional apnea at altitude, demonstrating that potential cardio-resistant adaptations to arrhythmogenesis exist across permanent HA populations. Acclimatized Lowlanders have further demonstrated an increased prevalence of arrhythmias at altitude.

## Introduction

Previous evidence has demonstrated that chronic exposure to low-oxygen environments [i.e., high-altitude (HA)] increases the prevalence of arrhythmic events in low-altitude dwelling populations. Such events have been previously documented in acclimatized Lowlanders during periods of elevated stress (i.e., physical exertion) above 4000 m (Woods et al., 2008; Behn et al., 2014; Boos et al., 2017). Recently, we demonstrated significant brady-arrhythmic events were present during a brief bout of apneic stress in Lowlanders at 5050 m (Busch et al., 2017), but not native Nepalese Sherpa. Whether the preservation of cardiac conduction in Sherpa exists amongst other HA population is unknown. Furthermore, it is unknown if such arrhythmic events seen in Lowlanders are evident in HA populations susceptible to chronic mountain sickness (CMS). Andeans have an overall shorter period of residence at altitude than other HA groups (Xing et al., 2008; Zhang et al., 2018) in addition to exhibiting differing physiological adaptation strategies of coping at altitude than Sherpa (Lahiri et al., 2000; Brutsaert et al., 2005; Beall, 2007; Hainsworth and Drinkhill, 2007). Furthermore, Andeans exhibit a higher prevalence of CMS compared with Sherpa (Villafuerte and Corante, 2016). We therefore investigated whether healthy (CMS-) and CMS afflicted (CMS +) Andeans exhibit abnormal cardiac conduction and ectopy.

## Methods

This study was a part of a multi-national research collaboration in the summer of 2018 to Cerro de Pasco, Peru (4330 m). The study design was approved by the Clinical Research Ethics Board of the University of British Columbia (H17-02687 and H18-01404), the University of Alberta Biomedical Research Ethics Board (Pro00077330), and the Universidad Peruana Cayetano Heredia Comité de Ética (number 101686). All participants gave written, informed consent prior to testing, and were provided a Spanish translator when necessary.

### Participant Recruitment

Seventeen high altitude native Andeans from Peru were recruited to participate in the study. Andeans without CMS (CMS-) and current CMS patients (CMS +; *N* = 8) were all local residents recruited from either Cerro de Pasco, Peru (4330 m), or from the surrounding villages. CMS + cutoff criteria included excessive erythrocytosis (Hb > 21g/dL) and current reported CMS symptoms [Qinghai CMS Score >6 (Leon-Velarde et al., 2005)]. CMS + were pre-recruited by a local physician prior to being recruited to participate. In addition, thirteen Lowlanders were tested within 3–8 days post-arrival at 4330 m. All Lowlander participants were members of the research expedition, of European background, and permanently resided near sea level. In addition, Lowlanders had not been above 2500 m within the 3 months prior to arrival at 4330 m. Though no physical activity patterns were not collected in either Lowlanders or Andeans, both Lowlanders and CMS- had no known history of cardiovascular or neurological disease. Two Lowlanders were reported as having mild acute mountain sickness (3 and 5 on the 2018 Lake Louise Acute Mountain Sickness Score (Roach et al., 2018). Both Lowlanders with reported mild AMS were included in the main analysis due to comparable HR and SpO2 to the main group.

### Study Design

All testing and data collection was performed at 4330 m. All participants abstained from caffeine, alcohol, and strenuous exercise for 12 h prior to testing. Participants were instrumented with an electrocardiogram (ECG, lead II; ADInstruments), Heart rate (HR) was calculated from the ECG R-R interval. Finger Pulse Oximetry (SpO_2_; Oximax N-600x, Medtronic), and beat-by-beat mean (MAP), systolic (SBP), and diastolic (DBP) pressures were calculated from the arterial pressure waveform that was calibrated against manual sphygmometer. Following instrumentation, basal ECG and cardiovascular function was measured during 10 min of quiet rest prior to attempting a volitional maximal end-expiratory apnea (to functional residual capacity). Prior to apnea, an investigator paced the participants’ breathing (2–3 breaths) to maintain rate and depth, while preventing hyperventilation. Participants were then instructed to hold their breath for as long as possible.

### Data and Statistical Analysis

HR, SpO_2_, and BP (Mean ± SD) was averaged during baseline (1 min pre-apnea attempt) and beat-by-beat for 10 cardiac cycles prior to volitional breakpoint for apneas. Nadir S_*p*_O_2_ and peak BP was obtained 10–20 s post-breakpoint. Electrophysiological characteristics [durations and intervals (Mean ± SD)] of the ECG were assessed during the last minute immediately preceding apneas; cardiac cycles (50–75) were over-laid, aligned with the R-wave and the aggregate was analyzed using automated software (Chart Pro 8.3.1). To account for variation in apnea duration, cardiovascular data from the final 10 cardiac cycles of each apnea were analyzed. A cardiologist (SD) identified and classified conduction abnormalities from ECG waveforms from the 3 beats immediately preceding and 3 beats following apnea breakpoint. HR, BP, and SpO_2_ was compared through pre-planned contrasts between CMS-, CMS + and Lowlanders (unpaired *T*-tests). An alpha adjustment (α’) for multiple comparisons (*c*) was performed by adjusting the *a priori* alpha (α, 0.05) using the experiment-wise error rate (α*_*e*_*) (Hinkle et al., 2003):

$$\alpha_{e}=1-{\left(1-\alpha \right)}^{c}$$

$$a^{\prime }=\frac{\alpha_{e}}{c}$$

Secondary analysis via one-way ANCOVA (SigmaPlot 13.3, Systat Software, Chicago, IL, United States) was used to control for duration at altitude in Lowlanders on the degree of bradycardia exhibited during apnea.

## Results

**FIGURE 1:**
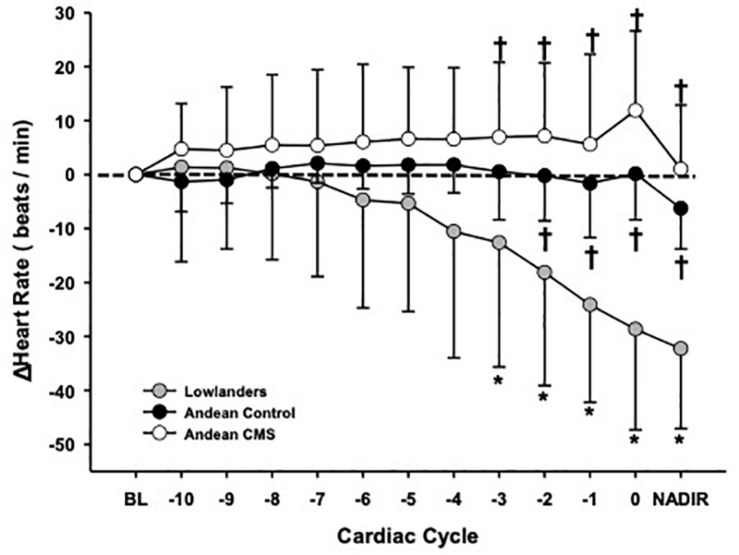
Absolute heart rate response (delta change) during maximal end-inspiratory apnea within Lowlanders (gray circle; N.13), Andean controls (CMS-: black circle; N.9), and Andean CMS patients (CMS +; white circle; N.8). The baseline HR for each group at 4330 m (represented as “0 beats/min”) and nadir response (lowest beats/min obtained during the last 10 cardiac cycles) have also been identified. Lowlanders developed significant bradycardia response prior to volitional breakpoint while both Andean controls and CMS patients had a preserved heart rate response. * Significant difference between cardiac cycle and baseline, *P* < 0.05. † Significant difference from Lowlanders, *P* < 0.05.

Participant characteristics, cardiovascular function, and ECG conduction abnormalities are outlined in Tables 1, 2. Participant HR responses during the last 10 cardiac cycles preceding apnea breakpoint are presented in Figure 1. Baseline HR and SpO_2_ was similar between CMS- (69 ± 8 beats/min; 80 ± 5%) and CMS + (62 ± 11 beats/min; 82 ± 1%). ECG analysis showed that CMS-/ + had comparable P-wave (169 ± 117 versus 102 ± 45 ms), PR- interval (239 ± 103 versus 184 ± 50 ms), QRS (75 ± 25 versus 85 ± 9 ms), and QTC (313 ± 91 versus 343 ± 35 ms) durations. One CMS + exhibited a Wandering atrial pacemaker during baseline. Apnea duration and SpO_2_ desaturation were similar between CMS- (23 ± 8 s; range 12–32 s; 78 ± 3%) and CMS + (33 ± 21 s; range 11–72 s; 74 ± 5%), while HR was preserved in both groups (−6 ± 1 and + 1 ± 12 beats/min; *p* = NS). CMS + and CMS- had lower MAP, SBP, and DBP at rest but the increase in pressure during apnea was similar between groups (Table 1). One CMS- developed a premature Atrial/Junctional escape prior to breakpoint (Table 2).

**TABLE 1:** Participant characteristics, cardiovascular function, and resting ECG metrics at rest and during maximal volitional apnea.

	**Lowlanders**	**Andeans**	
	***N* = 13 (Female = 4)**	**CMS− (*N* = 9)**	**CMS + (*N* = 8)**
**Participant characteristics**			
Age (years)	28 ± 7	45 ± 11†	38 ± 12†
Height (m)	1.74 ± 0.05	1.59 ± 0.04†	1.62 ± 0.05†
Weight (kg)	71 ± 7	68 ± 11	69 ± 12
Body mass index (kg/m^2^)	23.4 ± 2.1	26.8 ± 4.5†	26.1 ± 3.9†
**Resting cardiovascular function**			
Heart rate (beats/min)	77 ± 18	69 ± 8	62 ± 11†
SPO_2_ (%)	82 ± 3	80 ± 5	82 ± 1
Systolic blood pressure (mmHg)	128 ± 16	116 ± 12†	113 ± 7†
Diastolic blood pressure (mmHg)	81 ± 8	77 ± 9†	72 ± 4†
Mean arterial pressure (mmHg)	96 ± 10	90 ± 10†	86 ± 3†
**Resting ECG metrics**			
P-wave duration (ms)	100 ± 23	169 ± 117	102 ± 45
PR-Interval (ms)	161 ± 23	239 ± 103**†**	184 ± 50
QRS duration (ms)	79 ± 15	75 ± 25	85 ± 9
^▲^QTc (ms)	373 ± 77	313 ± 91	343 ± 35
**Cardiovascular response to apnea**			
Apnea duration (s)	13 ± 3	23 ± 8†	33 ± 21†
Heart rate (beats/min)	45 ± 10*	63 ± 11†	63 ± 15†
SPO_2_ nadir (%) ◆	79 ± 4*	74 ± 5*	78 ± 3*
Systolic blood pressure peak (mmHg) ◆	164 ± 20*	138 ± 16*†	137 ± 11*†
Diastolic blood pressure peak (mmHg) ◆	100 ± 6*	95 ± 8*	92 ± 9*†
Mean arterial pressure peak (mmHg) ◆	122 ± 9*	108 ± 9*†	108 ± 7*†

^▲^Framingham correction (QT + 0.154*(1-RR). ◆ Nadir/Peak response obtained from post-apnea breakpoint. * Significantly different from baseline; *p* < 0.05. † Significantly different from Lowlanders; *p* < 0.05.

**TABLE 2:** ECG Abnormalities in Andeans and Lowlanders at 4330 m at rest and during apnea.

	**Lowlanders**	**Andeans**	
	**(*N* = 13)**	**CMS− (*N* = 9)**	**CMS + (*N* = 8)**
**ECG abnormalities identified at rest**			
**Ectopy**			
Junctional rhythm	1	–	–
Wandering atrial pacemaker	1	–	–
**Conduction block**			
1° Atrio-ventricular block	1	–	1
ECG abnormalities identified during apnea**			
**Ectopy**			
Premature atrial contraction	–	1	1
Premature ventricular contraction	2	–	–
Ectopic atrial rhythm	1	–	-
**Sinus node dysfunction**			
Sinus pause/arrest	1	–	–
Sinus pause/arrest with junctional Escape	4	–	–
**Conduction block**			
1° Atrio-ventricular block	1	–	–
3° Atrio-ventricular block	1	–	–

ECG assessment was carried out by a cardiologist (SD) who was blinded to group and condition. ** Conduction abnormalities during apnea occur preceding or following (<3 beats) breakpoint. All measurements were taken using a standard lead II configuration.

**FIGURE 2:**
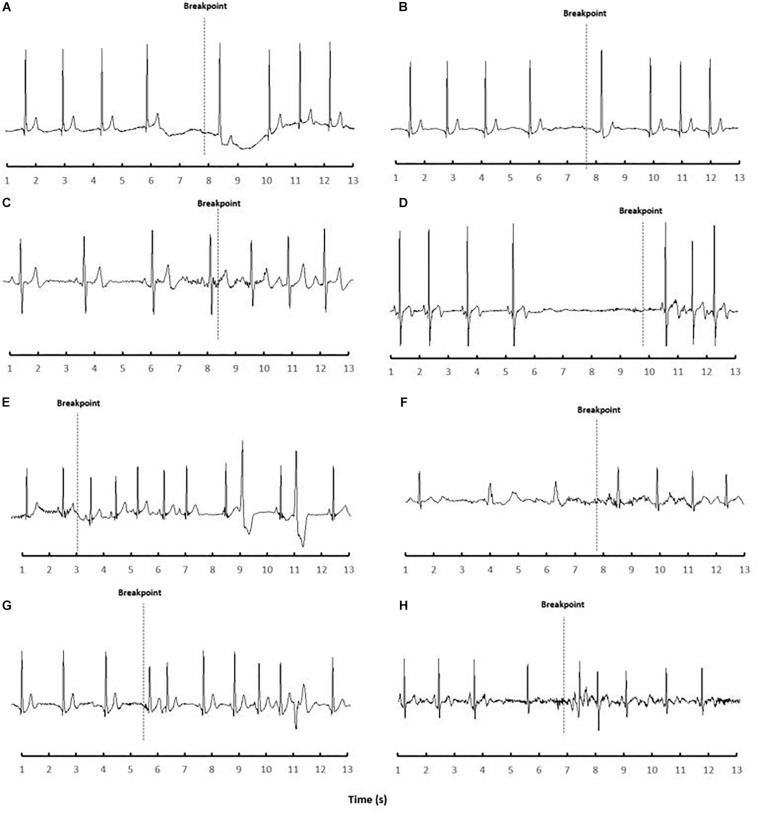
ECG recordings of Lowlanders identified with brady-arrhythmias during apnea (N.8) at 4330 m. Each frame **(A–H)** represents a single participant recording obtained preceding and/or proceeding volitional breakpoint. **(A)** sinus pause with junctional escape following volitional breakpoint; **(B)** ectopic atrial rhythm with junctional escape that began prior to volitional breakpoint and continued four beats following breakpoint; **(C)** premature atrial rhythm around volitional breakpoint; **(D)** sinus pause (4.3 s) immediately preceding volitional breakpoint; **(E)** ventricular bigeminy following breakpoint and 1st degree heart block (developed into 3rd degree); **(2F)** ventricular escape rhythm prior to volitional breakpoint; **(G)** premature ventricular contractions developed following breakpoint; **(H)** sinus pause with junctional escape preceding volitional breakpoint.

Lowlander baseline SpO_2_ (82 ± 1%) was similar to Andeans, while HR (77 ± 18 beats/min) was higher versus CMS + (*P* < 0.05). Resting ECG metrics in Lowlanders showed that P-wave (100 ± 23 ms), QRS (75 ± 25 ms), and QTC (373 ± 77 ms) durations were all similar to Andeans, while PR-Interval (161 ± 23 ms) was shorter in Lowlanders compared to CMS- (*P* < 0.05). Arrhythmias were detected in two Lowlanders during baseline, with one Lowlander exhibited a wandering atrial pacemaker with a 1**°**A-V block during baseline, while another showed a consistent junctional rhythm. Apnea attempts in Lowlanders (13 ± 3 s; range 11–20 s) were shorter than both CMS ± (*P* < 0.01), though Lowlander SpO_2_ nadir (79 ± 4%) was comparable. The post-apnea peak pressor response was higher in Lowlanders (MAP, and SBP; *P* < 0.01) than both CMS + /CMS- Andeans while DBP was only significantly lower in CMS + (*P* < 0.01). Lowlanders also showed significant bradycardia preceding breakpoint (−32 ± 15 beats/min; *P* < 0.001 versus baseline; *P* < 0.001 versus CMS-/CMS +). Prior to volitional breakpoint eight Lowlanders developed brief arrhythmic episodes (Figure 2). These arrhythmic events were categorized into either ectopic events [premature ventricular contractions (*N* = 2) and ectopic atrial rhythm (*N* = 1)], sinus node dysfunction [sinus pause/Arrest either with (*N* = 4) or without (*N* = 1) junctional escape], or atrio-ventricular conduction (A-V) blocks (*N* = 1). The one Lowlander with 1**°**A-V block during baseline also had a 1**°**A-V block during apnea that progressed to a 3**°**A-V block immediately preceding breakpoint.

## Discussion

This study reports that Andeans both with and without CMS exhibit a minimal incidence of arrhythmogenesis and preserved HR response to apneic stress at altitude. In addition, Andeans also exhibited a lower peak brachial pressure post-apnea and near doubling of apnea duration compared to Lowlanders, despite similar nadir SpO2 post-breakpoint. We believe these findings demonstrate that both ventilatory drive and cardiac vagal innervation was lower in Andeans during apnea. However, the absence of abnormal cardiac conduction during apnea in CMS + was striking given that CMS often promotes cardiac remodeling through a series of hypoxemia induced physiological adaptations including excessive erythrocytosis, hyperviscosity, impaired blood flow and pulmonary hypertension, eventual right atrial/ventricular hypertrophy, and altered ECG pattern (altered QRS complex/T-wave) (Dante and Arias-Stella, 2007; Xing et al., 2008; Villafuerte and Corante, 2016). Within our findings though there was no noted difference in QRS duration between Andeans and Lowlanders. Assessment of cardiac structure and function was not performed in the current study, which prevents us from confirming whether there were differences present between the healthy Andean participants and CMS patients. One consideration in our CMS + group was that the majority of participants displayed only mild CMS symptoms, with comparable oxygen saturation to both healthy Andeans and partially acclimatized Lowlanders, regardless of the evident excessive erythrocytosis. We therefore cannot comment on whether arrhythmias become evident under more severe cases of CMS.

The minimal incidence of brady-arrhythmias and preserved HR response in Andeans are consistent with our previous findings from indigenous Nepalese Sherpa (Busch et al., 2017) and further demonstrates an absence of vagal-induced arrhythmogenesis in HA populations. However, the current study also showed Andeans had overall longer apnea durations than Sherpa. As both groups performed maximal apnea attempts at functional residual capacity, the difference between groups may in part be explained through Andeans exhibiting a reduced ventilatory drive (via blunted chemoreflex sensitivity) to hypoxia stimuli (Severinghaus et al., 1966; Beall, 2007). We believe the peripheral chemoreceptors plays a considerable role in the genesis of significant sympatho/vagal conflict to the heart (Busch et al., 2017). Previous publications also demonstrate ventilatory drive differs between highland groups (Beall et al., 1997; Brutsaert et al., 2005), with Andeans often demonstrating a blunted ventilatory response. Furthermore, progressive changes in carotid body morphology (Arias-Stella and Valcarcel, 1976; Lahiri et al., 2000) suggest gradual chemoreflex desensitization overtime in Andeans. However, one consideration of the current study is that HR variation prior to volitional breakpoint was similar between Sherpa and Andeans which contradicts differences in ventilatory drive observed each highlander group. Though outside the scope of the current study, there may be differential regulation between ventilatory and cardiac autonomic control in highlander groups which is not explained through chemoreflex sensitivity. Furthermore, it is unknown whether the absence of apnea-induced arrhythmias in HA populations are due to an overall lower degree of cardiac sympatho/vagal innervation to the heart, or alterior underlying physiological mechanisms that are specifically cardio-protective to altered vagal drive. Although Andeans and Sherpa exhibited similar HR responses and an absence of arrhythmogenesis during apnea, the underlying physiological mechanisms potentially differ between HA populations. Further investigation is therefore required to confirm the current findings in other HA residing populations (i.e., Native Ethiopian Highlanders) alongside the underlying physiological differences that cause an absence of arrhythmogenesis under apneic stress.

The presence of significant brady-arrhythmic events further demonstrate that Lowlanders exhibit abnormal cardiac conduction when under apneic stress at altitude. These events do not appear to be of a baroreflex origin as brachial pressure increased similarly in Lowlanders and Andeans during apnea, though Lowlanders had an overall larger increase in post-apnea peak BP than Andeans. We postulate that the brady-arrhythmic responses in Lowlanders during apnea are attributed to conflicting sympathetic/vagal co-innervation (Shattock and Tipton, 2012) that is compounded with both altitude-induced sympathetic hyperactivity (Hansen and Sander, 2003; Lundby et al., 2017; Simpson et al., 2019) and pre-existing hypoxic stress. This conflict likely becomes more evident through progressive chemoreflex hypersensitivity during longer-term residency at altitude (Dempsey et al., 2014) and ascent. Previously, supplemental oxygen (FiO_2_ 1.00) has abolished these events during apnea at 5050 m (Busch et al., 2017), demonstrating the peripheral chemoreceptors likely play a considerable role. However, any considerable autonomic stress (Woods et al., 2008; Boos et al., 2017) appears to be capable of evoking arrhythmogenesis independent of significant chemoreflex activation, though further research is required. Although these events were benign in healthy Lowlanders, future direction should be focused on clinical populations at greater risk of adverse complications arising from arrhythmogenesis at altitude, including conditions where consistent cardiac vagal innervation may occur such as sleep disordered breathing (i.e., Cheyne-Stokes respiration) (Cummings and Lysgaard, 1981) commonly observed at altitude.

### Considerations and Limitations

This study has several considerations that should be acknowledged. As previously mentioned there was no assessment of cardiac structure and function independent of resting ECG metrics in the current study, which limits our ability to determine whether any cardiac abnormalities existed in CMS patients. This would be an important consideration for future expeditions to pursue in order to determine if those exhibiting impaired cardiac function show a higher prevalence of brady-arrhythmias. Another consideration is participant recruitment, with Lowlanders consisting of research members involved in the expedition and all Andeans being locally recruited from around Cerro de Pasco. Due to the constraints of an expedition setting Lowlanders were tested on days 3–8 post-arrival at 4330 m. As of this study, the exposure duration and minimum altitude necessary to promote arrhythmogenesis is not currently known, though previous findings suggest that they are more likely during the transition from acute hypoxia exposure to longer duration travel at altitudes above 4000 m (Cummings and Lysgaard, 1981; Woods et al., 2008; Behn et al., 2014; Brooks et al., 2017; Busch et al., 2017). Though the duration of the testing period in the current study aligns with the testing periods of Lowlanders from our previous work (Busch et al., 2017), the time course for the development of conduction abnormalities will need to be addressed on future expeditions.

One final consideration is the noted age difference between Lowlanders and Andeans. Previous findings (Behn et al., 2014) suggest that younger low-altitude dwelling individuals may be more likely to develop arrhythmias at altitude due to greater underlying vagal drive. Though Lowlanders in the current study were on average younger than Andeans, we do not believe that age played a significant factor in our results. Not only was the degree of bradycardia markedly greater in Lowlanders prior to volitional breakpoint than Andeans (see Figure 1 and Supplementary Figure S1), but an additional *post hoc* ANCOVA incorporating age as a covariate still noted differences in the degree of bradycardia between groups during apnea. Furthermore, arrhythmias were not solely present in our (younger) lowlander population, as one Lowlander (age 50) and one EE- (age 62) who developed arrhythmia prior to breakpoint (sinus pause with junctional escape and premature atrial beat, respectively). Both individuals being the oldest of their respective groups. Finally, the current findings support our conclusion that Highlanders do not exhibit brady-arrhythmias, given almost all of Andean participants, including younger individuals, exhibited a minimal incidence of brady-arrhythmias compared to a high occurrence in Lowlanders.

In summary, these findings demonstrate Andeans with or without mild CMS exhibit an absence of arrhythmic events that is akin to Nepalese Sherpa, though the underlying physiological mechanisms remain to be uncovered. It is important to note though that the absence of arrhythmia in Andean CMS + patients would merit from further investigation into more severe cases of CMS. Regardless, the absence of such events may be beneficial to surviving at high altitude, as Lowlanders exhibit a greater risk of developing abnormal cardiac conduction at altitude under a variety of stressors at altitude. In future research expeditions we recommend that in addition to studying the possible duration factor on when arrhythmogenesis develops in Lowlanders, other interventions be explored relative to travel at altitude that might uncover arrhythmias.

## Data Availability Statement

The datasets generated for this study are available on request to the corresponding author.

## Ethics Statement

The studies involving human participants were reviewed and approved by 1.) Clinical Research Ethics Board of the University of British Columbia 2.) University of Alberta Biomedical Research Ethics Board 3.) Universidad Peruana Cayetano Heredia Comité de Ética. The patients/participants provided their written informed consent to participate in this study.

## Author Contributions

LS, GV-G, FV, PA, MT, JM, MS, and CS conceived and contributed to the research study design. AS, VM, LS, RF-M, JM, MS, and CS performed the experiments and data collection. SB, SD, and CS analyzed and interpreted the data and involved in statistical analysis. SB prepared the figures and manuscript. SD, PA, MT, JM, MS, and CS edited and revised the manuscript. All authors approved the submitted version of the manuscript.

## Conflict of Interest

The authors declare that the research was conducted in the absence of any commercial or financial relationships that could be construed as a potential conflict of interest.
