# Supplementary material for: Global REACH: Assessment of Brady-Arrhythmias in Andeans and Lowlanders During Apnea at 4330 m
Source: Front Physiol. 2020 Jan 22;10:1603. doi: 10.3389/fphys.2019.01603 (PMC6987448; doi:10.3389/fphys.2019.01603)
Supplement: Supplementary file 1 [file Table_1.DOCX]

**A**

**B**


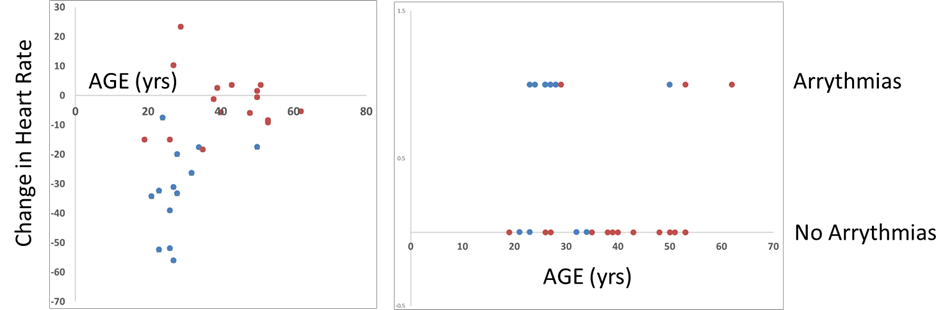


**Supplemental Figure 1.** Graphical representation of the change in heart rate during apnea (Panel A) and occurrence of arrhythmias during apnea (Panel B - grouped at a value of 1) with regards to participant age (X-axis).  Individual Lowlander data points are depicted in blue while Andean Quechua are red.  Panel A shows that younger Andeans do not exhibit the same bradycardia as similar aged Lowlanders pre-volitional breakpoint, with one older Lowlander participants also demonstrating a larger bradycardia response than similar aged Andeans. Panel B shows that incidence of arrhythmias in Lowlanders and Andeans was not solely observed in younger participants, with older individuals also exhibiting brady-arrhythmias during apnea.
